# Elevated Blood Pressure and Serum γ -Glutamyltransferase as Significant Characteristics of Smokers With Chronic Kidney Disease

**DOI:** 10.5812/numonthly.20746

**Published:** 2014-07-05

**Authors:** Yuka Noborisaka, Masao Ishizaki, Michiko Yamazaki, Ryumon Honda, Yuichi Yamada

**Affiliations:** 1 Department of Social and Environmental Medicine, Kanazawa Medical University School of Medicine, Uchinada, Japan

**Keywords:** Smoking, Chronic Kidney Disease, Blood Pressure, Serum gamma-glutamyltransferase, Insulin resistance, Inflammation

## Abstract

**Background:**

Smoking is a risk factor for chronic kidney disease (CKD). However, it is speculated that only a small subset of sensitive smokers develop CKD.

**Objectives:**

We aimed to reveal the characteristics of such smokers sensitive to the renal effects of smoking with respect to cardiovascular (CV) risk factors associated with smoking and/or CKD.

**Patients and Methods:**

Renal functions and CVD risk factors were assessed in middle-aged male workers. The patients were comprised of 336 nonsmokers, 332 smokers currently smoking up to one pack per day, and 38 who smoked more than one pack per day. CKD was determined by estimated glomerular filtration rate (eGFR) from serum creatinine and urinary albumin to creatinine ratio (ACR). The independent and interactive effects of smoking and CKD on the CVD risk factors adjusted for age, body mass index, alcohol consumption, and physical activity were statistically analyzed.

**Results:**

In comparison to nonsmokers, smokers had significantly higher waist circumference, white blood cells (WBC), serum triglycerides, γ-glutamyltransferase (GGT), and C-reactive protein (CRP) and lower serum high-density lipoprotein cholesterol and uric acid. On the other hand, blood pressure (BP) and WBC tended to be higher in those showing CKD than others. Serum GGT and fasting plasma glucose were significantly higher, and insulin resistance index of homeostatic model assessment (HOMA-IR) tended to be higher in those with CKD. Serum CRP was especially high in those with moderate to severe CKD. A significant interactive effect of smoking and CKD on BP and serum GGT levels was detected, i.e. BP and GGT were not different in the subjects among nonsmokers with and without CKD, but were conspicuously high among smokers with CKD. No significant interactive effect was found on either HOMA-IR or serum CRP.

**Conclusions:**

Smokers with a higher BP and/or serum GGT may be at a higher risk of developing CKD. The associations of BP and serum GGT with CKD in smokers are not entirely mediated by increased insulin resistance or chronic inflammation caused by smoking.

## 1. Background

Cigarette smoking is an independent risk factor of developing chronic kidney disease (CKD) in the general population (1). Since smokers in the general population often show a higher glomerular filtration rate (GFR) and only a small subset of smokers develop CKD, Yoon et al. (2) speculated that only those who are extremely sensitive to the renal toxic effects of smoking would develop CKD while the majority of smokers would retain normal renal function throughout their lives. They recommended defining these subsets to prevent smoking-related CKD in the general population.

## 2. Objectives

The aim of the present study was to reveal the characteristics of patients sensitive to the adverse effects of smoking who were more susceptible to develop CKD with respect to cardiovascular (CV) risk factors associated with smoking and CKD.

## 3. Patients and Methods

The subjects were comprised of 923 male workers aged 30 to 64 years from an electronic devices producing factory who underwent annual health check-ups including measurements of the serum creatinine (Cr) concentration. Written informed consent was obtained from all the subjects, and the aim and design of this study were approved by the Ethics Committee of Kanazawa Medical University.

### 3.1. Data Collection

The subjects were asked to fast overnight and to refrain from smoking 30 minutes before starting the health check-up. Height and body weight were measured without jackets and shoes, and 1.0 kg was subtracted to obtain the net weight. Body mass index (BMI) was calculated as the net body weight (kg) divided by the squared height (m). Waist circumference (cm) was measured at the navel level. Blood pressure (BP) was measured after resting on a chair for five minutes or longer using an electronic sphygmomanometer with an appropriate size cuff with arm maintained at the heart level. For the convenience of the statistical analysis, mean BP was calculated by the following formula: 

[Diastolic BP + (systolic BP - diastolic BP)]/3.

Fasting venous blood samples were analyzed for hematological tests including white blood cell count (WBC) and biochemical parameters including serum concentrations of low density lipoprotein and high density lipoprotein cholesterol (LDLc and HDLc, respectively), triglycerides (TG), uric acid (UA), Cr, and the activities of hepatic enzymes including γ-glutamyltransferase (GGT). The concentrations of fasting plasma glucose (FPG), serum insulin, and high-sensitivity C-reactive protein (CRP) were also measured. Homeostasis model assessment (HOMA) indices of insulin secretion (HOMA-β) and insulin resistance (HOMA-IR) were calculated from FPG and serum insulin. Spot urine samples collected during the health check-ups were measured for albumin excretion, i.e. urinary albumin to creatinine ratio (ACR).

The data of alcohol and cigarette consumption and physical activity at leisure times were collected in the health check-ups using a questionnaire. Current smoking habit was classified into four categories: nonsmokers; ex-smokers; moderate smokers (consuming up to one pack per day); and heavy smokers (consuming more than one pack per day). Usual alcohol consumption was categorized as follows: nondrinkers, who drank a very small amount only a couple of times a year; mild drinkers, who consumed alcohol more often than once a month but less than 30 mL/day on average; moderate drinkers, who consumed 30 to 59 mL/day on average; and heavy drinkers, who consumed 60 mL/day or more. Significant physical exercise was defined as being more intense than brisk working and lasting for 30 minutes or longer. The subjects were defined to be physically “inactive” when they performed physical exercise less than once a month, “mildly active” when they performed exercise more often but less than once a week, “moderately active” when they performed it once or twice a week, and “active” when they performed it more than twice a week. The four categories of alcohol consumption and physical activity were scored from zero to three for the statistical analysis.

### 3.2. Definition of Severity of Chronic Kidney Disease

GFR was estimated from age, sex, and serum Cr concentration (eGFR) using the formula proposed by the Japanese Society of Nephrology (3), and classified into six categories from G1 through G5 as follows: G1 (eGFR of 90 or higher), G2 (60 to 89), G3a (45 to 59), G3b (30 to 44), G4 (15 to 29), and G5 (less than 15 mL/min/1.73 m^2^), consecutively. 

Urinary ACR was defined as A1 (normal; ACR < 30 mg/gCr), A2 (microalbuminuria; ACR of 30-299 mg/gCr), and A3 (macroalbuminuria; ACR ≥ 300 mg/gCr). The subjects were defined as free of CKD when they had eGFR of G1 or G2 with normal amounts of albumin in urine (A1), mild CKD when eGFR was G1 or G2 with microalbuminuria (A2) or G3a with A1, moderate CKD when eGFR was G1 or G2 with macroalbuminuria (A3), or G3a with A2, or G3b with A1, and severe CKD when they had eGFR of G3a with A3, or G3b with A2 or A3, or G4 or G5 regardless of albuminuria (4).

### 3.3. Statistical Analysis

The means of CVD risk factors measured in the health check-ups were compared in the categories of cigarette consumption and CKD. The differences in the means were tested by ANOVA and by a generalized linear model (GLM) analysis adjusting for age, BMI, and the scores of alcohol consumption as well as physical activity. Paired comparisons were conducted by a least square method (LSM). Then interactive effects of cigarette consumption and CKD, as well as independent effects on the means of the CVD risk factors were analyzed by a GLM method adjusting for these confounders. All the statistical analysis were conducted using SPSS ver. 19 distributed by IBM.

## 4. Results

### 4.1. Smoking Habits, Renal Function and Cardiovascular Risk Profile

The subjects consisted of 13 workers aged 30 years, 31 aged 35 years, and 879 aged 40 to 64 years (mean age, 46.4 years); there were 336 (36.4%) nonsmokers, 217 (23.5%) ex-smokers, 332 (36.0%) mild smokers, and 38 (4.1%) heavy smokers.

The matrix of G (eGFR) and A (urinary ARC) distributions in the subjects were as follows: 200 with G1 (A1, 186; and A2, 14), 697 with G2 (A1, 659; A2, 36; and A3, 2), 24 of G3a (A1, 24), and two of G3b (A1, 1; and A2, 1). None of the subjects showed depressed eGFR corresponding to G4 or G5. Consequently, the prevalence of CKD was 78 (8.5%) of the total subjects; 74 (8.0%) showing mild CKD, 3 (0.3%) moderate CKD, and 1 (0.1%) severe CKD. Among 336 nonsmokers, 45 (8.1%) showed mild, 1 (0.2%) moderate, and 1 (0.2%) severe CKD. Among 217 ex-smokers, 45 subjects (8.1%) showed mild, 1 (0.2%) moderate, and 1 (0.2%) severe CKD. On the other hand, 24 patients (7.2%) with mild and 1 (0.3%) with moderate CKD were found in the 332 mild smokers. Among 38 heavy smokers, 5 (13.2%) showed mild and 1 (2.6%) moderate CKD. The prevalence rate of CKD in the heavy smokers was 15.8%, which was nearly twice as high as the rate in the nonsmokers.

In the present subjects, the ex-smokers were distinct from the nonsmokers with respect to the profile of CVD risk factors, i.e. they showed the highest BMI, waist circumference, mean BP, and serum TG among the four categories of cigarette consumption while the nonsmokers showed the lowest respective values. On the other hand, regarding urinary ACR or eGFR, the ex-smokers showed similar results to the nonsmokers. Therefore, the 217 ex-smokers could not be regarded simply the same as nonsmokers and therefore, were excluded in the consequent statistical analysis.

### 4.2. The Associations of Smoking, Chronic Kidney Disease and Cardiovascular Risk Factors

Table 1 shows the means and standard errors (SEs) of the CVD risk factors, as well as eGFR and urinary ACR, adjusted for age, BMI, alcohol consumption, and physical activity in the nonsmokers and mild and heavy smokers. As shown in the table the means of eGFR increased in parallel with the increase in the number of cigarettes consumed, while urinary ACR was significantly higher in the mild smokers than in the nonsmokers. As for the CVD risk factors, waist circumference in the mild smokers and WBC in the heavy smokers were significantly higher than those of nonsmokers. Serum LDLc tended to be lower in the heavy smokers than mild smokers. Serum HDLc was significantly lower and TG was significantly higher in the mild smokers than in nonsmokers. Serum GGT was significantly higher in the mild and heavy smokers than in nonsmokers. Serum UA was significantly lower in the heavy smokers. Serum CRP was significantly higher in the mild smokers than nonsmokers. No significant differences were found in FPG, serum insulin, or HOMA indices among patients regarding smoking status.

Table 2 illustrates the means and SEs of the CVD risk factors in the subjects assigned to the three categories of CKD, i.e. 649 subjects being free of CKD, 53 with mild CKD, and four with moderate or severe CKD. Waist circumference tended to be smaller in the subjects with moderate or severe CKD in comparison with those with mild CKD. Mean BP was higher in those showing CKD than in those free of CKD. Although WBC was higher in the subjects with mild CKD than in those free of CKD, it was lower in those with moderate or severe CKD in comparison with those with mild CKD. Serum LDLc was significantly lower in those with CKD as compared to those free of CKD. Neither serum HDLc nor TG showed significant differences between those with and without CKD. Serum GGT was significantly higher in those with mild CKD. FPG showed progressively higher mean values with the progression of CKD. Although serum insulin levels were significantly higher in those with mild CKD in comparison with those free of CKD, they were lower in those with moderate to severe CKD than in those free of CKD or with mild CKD; however, the differences were not significant. HOMA-β was significantly lower in those with moderate or severe CKD in comparison with those free of CKD or with mild CKD. HOMA-IR was higher in those with mild CKD than in those free of CKD. Serum CRP was higher in those with moderate or severe CKD in comparison with that of other patients.

**Table 1.** Means and Standard Errors of Cardiovascular Diseases Risk Factors and Renal Function in the Subjects Regarding Cigarette Consumption ^a,b^

	A. Nonsmokers		B. Smoke up to 1 Pack/day		C. Smoke > 1 Pack/day		Statistical Significance ^d^		
	N = 336 ^c^		N = 332		N = 38				
CVD Risk factors	M	SE	M	SE	M	SE	A vs B	A vs C	B vs C
**Waist circumference, cm**	82.7	0.19	83.3	0.19	83.7	0.57	P < 0.05	ns	ns
**Mean BP, mmHg**	90.7	0.56	89.7	0.56	88.4	1.68	ns	ns	ns
**WBC, × 10** ^**3**^ **/cm** ^**2**^	6.31	0.10	6.49	0.10	6.97	0.29	ns	P < 0.05	ns
**Serum LDL ** ^**e**^ **, mg/dL**	125.7	1.73	127.1	1.73	118.0	5.19	ns	ns	#
**Serum HDL ** ^**e**^ **, mg/dL**	61.9	0.73	58.4	0.73	57.9	2.18	P < 0.01	P < 0.10	ns
**Serum TG, mg/dL ** ^**e**^	95.9	1.03	108.7	1.03	110.0	1.08	P < 0.01	ns	ns
**Serum GGT, U/L ** ^**e**^	32.6	1.03	36.4	1.03	40.2	1.10	P < 0.05	P < 0.05	ns
**Serum UA, mg/dL**	5.93	0.06	5.86	0.06	5.46	0.19	ns	P < 0.05	P < 0.05
**FPG, mg/dL**	96.7	0.84	96.1	0.84	99.5	2.52	ns	ns	ns
**Serum insulin, U/mL ** ^**e**^	6.27	1.03	6.10	1.03	5.82	1.08	ns	ns	ns
**HOMA-β ** ^**e**^	71.3	1.03	71.1	1.03	64.9	1.08	ns	ns	ns
**HOMA-IR ** ^**e**^	1.48	1.03	1.43	1.03	1.40	1.09	ns	ns	ns
**Serum CRP, × 10** ^**-2 **^ **U/mL ** ^**e**^	2.31	1.06	3.03	1.06	3.16	1.20	P < 0.01	ns	ns
**eGFR, mL/min/1.73 m** ^**2**^	78.7	0.62	83.9	0.62	89.1	1.87	P < 0.001	P < 0.001	P < 0.01
**Urinary ACR, mg/gCr ** ^**e**^	5.85	1.04	7.38	1.04	7.31	1.14	P < 0.001	ns	ns

^a^ Data are adjusted for age, body mass index, alcohol consumption, and physical activity.

^b^ Abbreviations: CVD, cardiovascular diseases; BP, blood pressure; WBC, white blood cells; LDL, low-density lipid; HDL, high-density lipid; TG, triglyceride; GGT, γ-glutamyltransferase; UA, uric acid; FPG, fasting plasma glucose; HOMA-β, homeostasis model assessment indices of insulin secretion; HOMA-IR, homeostasis model assessment indices of insulin resistance; CRP, C-reactive protein; eGFR, estimated glomerular filtration rate; and ACR, Albumin to creatinine ratio.

^d^ The results of GLM analysis and paired comparison with LSM. ns, P ≥ 0.10.

^c^ Numbers of the subjects.

^e^ Geometric mean and geometric standard error.

**Table 2.** Means and Standard Errors of Cardiovascular Diseases Risk Factors in the Subjects Regarding the Criteria of CKD Severity ^a,b^

	A. Free of CKD		B. Mild CKD		C. Moderate/Severe CKD ^c^		Statistical Significance ^e^		
	N = 649 ^d^		N = 53		N = 4				
	M	SE	M	SE	M	SE	A vs B	A vs C	B vs C
**Waist Circumference, cm**	83.0	0.14	83.8	0.49	80.4	1.77	ns	ns	P < 0.10
**Mean BP, mmHg**	89.9	0.40	92.6	1.42	98.9	5.18	P < 0.10	P < 0.10	ns
**WBC, × 10** ^**3**^ **/cm^2^**	6.40	0.07	6.88	0.25	5.19	0.90	P < 0.10	ns	P < 0.10
**Serum LDL **^**e**^, ** mg/dL**	127.0	1.23	115.4	4.37	94.4	15.9	P < 0.05	P < 0.05	ns
**Serum HDL **^**e**^, ** mg/dL**	59.8	0.52	63.0	1.86	54.5	6.79	ns	ns	ns
**Serum TG, mg/dL** ^**f**^	101.7	1.02	113.0	1.08	113.5	1.29	ns	ns	ns
**Serum GGT, U/L ** ^**f**^	34.2	1.02	41.4	1.08	43.3	1.34	P < 0.05	ns	ns
**Serum UA, mg/dL**	5.85	0.05	6.07	0.16	6.78	0.59	ns	ns	ns
**FPG, mg/dL**	95.8	0.59	103.9	2.10	123.5	7.65	P < 0.001	P < 0.001	P < 0.05
**Serum Insulin, U/mL ** ^**f**^	6.12	1.02	6.67	1.07	5.48	1.26	P < 0.05	ns	ns
**HOMA-β ** ^**f**^	71.1	1.02	68.4	1.07	41.7	1.27	ns	P < 0.05	P < 0.05
**HOMA-IR ** ^**f**^	1.44	1.02	1.66	1.08	1.59	1.29	P < 0.10	ns	ns
**Serum CRP, × 10** ^**-2**^ ** U/mL ** ^**f**^	2.60	1.04	3.36	1.17	10.8	1.77	ns	P < 0.05	P < 0.05

^a^Data are adjusted for age, body mass index, alcohol consumption, and physical activity.

^b^Abbreviations: CKD, chronic kidney disease; CVD, cardiovascular diseases; BP, blood pressure; WBC, white blood cells; LDL, low-density lipid; HDL, high-density lipid; TG, triglyceride; GGT, γ-glutamyltransferase; UA, uric acid; FPG, fasting plasma glucose; HOMA-β, homeostasis model assessment indices of insulin secretion; HOMA-IR, homeostasis model assessment indices of insulin resistance; and CRP, C-reactive protein.

^c^ The criteria of CKD severity followed KDIGO report.

^e^ The results of GLM analysis and paired comparison with LSM. ns, P ≥ 0.10.

^d^ Numbers of the subjects.

^f^ Geometric mean and geometric standard error.

### 4.3. The Interactive Effect of Smoking and Chronic Kidney Disease on Cardiovascular Diseases Risk Factors

**Figure 1.**
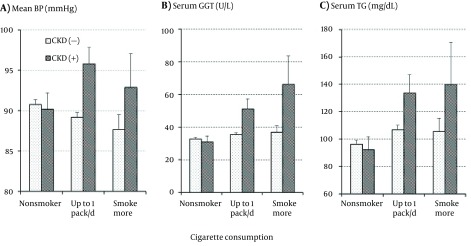
Means and Standard Errors of Mean Blood Pressure (a), Serum γ-Glutamyltransferase Activity (b), and Serum Triglyceride Concentration (c) in the Subjects With and Without Chronic Kidney Disease According to Cigarette Consumption.

The interactive effect of smoking and CKD as well as their independent effects on the CVD risk factors were further analyzed. A significant interactive effect was found in the levels of mean BP and serum GGT as shown in Figure 1A and 1B, respectively. Mean BP and serum GGT did not differ between those with and without CKD or were even somewhat lower in those with CKD as long as the subjects were nonsmokers; however, both mean BP and serum GGT were much higher in those with CKD than in those without CKD in the smokers. With increasing cigarette consumption in the subjects free of CKD, mean BP decreased while serum GGT increased. The association of serum GGT with smoking and CKD was similar to that of serum TG with smoking and CKD as shown in Figure 1 C, but the interactive effect of smoking and CKD did not reach a significant level.

**Figure 2.**
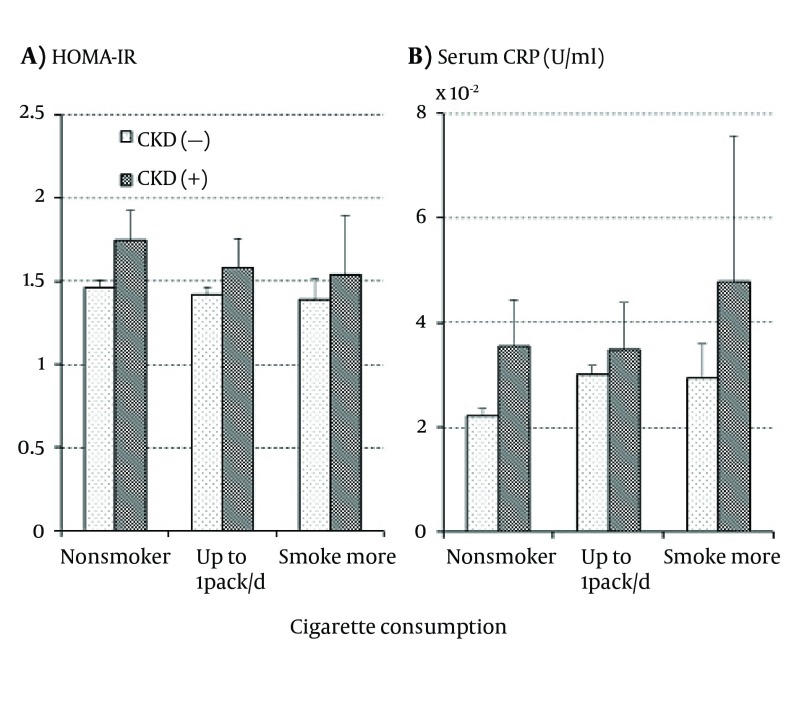
The Means and Standard Errors of HOMA-IR (a) and Serum C-Reactive Protein Concentration (b) in the Subjects With and Without Chronic Kidney Disease According to Cigarette Consumption.

As shown in Figure 2 A, HOMA-IR was higher in those with CKD than in those free of CKD regardless of cigarette consumption while it decreased with increasing cigarette consumption regardless of CKD presence. None of the effects of smoking, CKD, and the interaction between smoking and CKD were, however, statistically significant. On the other hand, Figure 2B showed that serum CRP increased with increasing cigarette consumption regardless of CKD presence. The increase in CRP was somewhat more marked in those with CKD than in those without it and a similar association was observed in WBC; however, no significant independent or interactive effects of smoking and CKD were detected on either serum CRP or WBC.

## 5. Discussion

Markedly higher BP and serum GGT were observed in smokers with CKD than in those free of CKD (Figure 1), while both were similar in nonsmokers with and without CKD. Statistical analysis showed a significant interactive effect of smoking and CKD on the BP and serum GGT levels. Although the interactive effect was not significant, a similar association of serum TG with smoking and CKD was observed as shown in Figure 1C. These findings are reasonable since high BP (5-7), serum TG (8, 9), and GGT (10, 11) have been reported to predict the future development of CKD. Therefore, although this cross-sectional observation did not provide any clue to the causal association, the present results suggested that those with a higher BP, serum TG, and/or serum GGT comprised a high-risk population for the development of smoking-related CKD.

Prolonged hypertension undoubtedly accelerates the deterioration of renal function resulting in CKD. However, it remains uncertain why BP elevations in CKD patients were conspicuous only in smokers but not in nonsmokers since BP in smokers is not necessarily higher than that in nonsmokers as shown in the present and previous studies (12). Smoking a cigarette is known to cause a temporary rise of BP mainly due to stimulation of the renin-angiotensin system (RAS) by nicotine in tobacco smoke (13, 14). However, BP was usually measured in clinics or health screenings with a considerable interval since the most recent cigarette, and such BP measurements may be misleading by showing a lower BP than usual. On the other hand, smokers tended to show a higher BP at ambulatory monitoring (15, 16). Therefore, BP would be elevated by habitual smoking probably via stimulated RAS, chronic inflammation, or increased insulin resistance (17) although it may be often masked and promote the development of CKD.

Our previous study demonstrated that elevated serum GGT played a certain role in the development of CKD in smokers (18) although the mechanisms underlying the association between elevated serum GGT and CKD in smokers have remained uncertain. GGT catalyzing extracellular glutathione (GSH) and plays a role in maintaining GSH content in cells, a major antioxidant substance, while it may promote oxidative stress producing free radicals or super-oxides during the catalysis of extracellular GSH (19). It is not likely, however, that the elevated levels of oxidants caused by elevated serum GGT activity directly promote the development of CKD since those oxidants, if present, are readily removed by antioxidant substances abundant in blood (20). The more plausible mechanisms are increased insulin resistance and/or chronic inflammation induced by smoking, which are known to play central roles in the development of CKD (21, 22).

In the present results, as shown in Table 2 and Figure 2 A, HOHA-IR was higher in the subjects with CKD than in those free of CKD in both smokers and nonsmokers, suggesting that the effects of increased insulin resistance on the development of CKD are independent of smoking. On the other hand, HOMA-IR decreased with increasing cigarette consumption, which was contrary to the previous findings of increased insulin resistance in smokers (23, 24). The reasons for this discrepancy are not clear and the association between smoking and HOMA-IR has not been consistent in previous studies (25, 26). It is possible that insulin resistance measured by glucose clamp or steady-state plasma glucose methods gives results different from HOMA-IR estimated from FPG and serum insulin. In the present study, the association of HOMA-IR with smoking and CKD was different from those of BP and serum GGT in all cases. Therefore, insulin resistance, as far as it was measured by HOMA-IR, did not seem to mediate the association between BP and serum GGT elevations with the development of CKD in smokers.

In contrast to HOMA-IR, serum CRP increased with the increase in cigarette consumption as shown in Table 1 and Figure 2B, which was consistent with previous studies (27, 28); however, the increases were similar between the subjects with and without CKD and thus, the interactive effect of smoking and CKD on serum CRP was not significant. A similar association with smoking and CKD was observed in WBC, another inflammatory index. These results suggest that chronic inflammation is associated with the development of CKD independent of cigarette smoking although smoking would cause chronic inflammation. Summing up, the present study suggests that neither insulin resistance nor chronic inflammation entirely mediated the association of smoking with the elevations of BP and serum GGT as well as the development of CKD. Other mechanisms might mediate the associations.

Although it was not investigated in the present study, another possible mechanism is oxidative stress caused by smoking. Tobacco smoke contains various oxidants (29), and smokers often showed elevations of oxidative stress markers such as F2-isoprostanes, thiobarbituric acid-reactive substances, and 8-hydroxydeoxyguanosine in the blood (30, 31) and 8-epi-prostaglandin F2α in the urine (32). On the other hand, serum GGT was observed to correlate with oxidative stress markers or antioxidant substances in the blood and urine (33, 34). Therefore, although direct effects of elevated serum GGT on the elevation of oxidative stress are unlikely (20), the elevated BP and serum GGT may reflect elevated oxidative stress in smokers. Further investigations are required to elucidate the role of oxidative stress in the association of smoking with BP and serum GGT elevations and the development of CKD.

As for the other CVD risk factors, it has been demonstrated that smoking causes dyslipidemia characterized by a higher serum TG and a lower serum HDLc; elevated serum TG, has been identified as a predictive factor for the future development of CKD (8, 9). In addition, CKD has been shown to cause considerable changes in the serum lipids profile (35). In the present study, as shown in Tables 1 and 2 Figure 1C, serum TG was markedly elevated with increase in cigarette consumption in the subjects with CKD; it suggests a not-fully confirmed but possible role in the development of CKD in smokers, which should be further investigated. On the other hand, although not shown in the figures, decreases in HDLc with cigarette consumption were obvious in the subjects without CKD but not in those with CKD. Statistical analysis showed no significant effects of smoking, CKD, and their interaction on serum HDLc levels. Thus, the role of depressed serum HDLc in the development of CKD in smokers remains unclear. 

Hyperuricemia has been demonstrated to predict the development of CKD (36, 37). In the present study, as shown in Table 2, serum UA was elevated with the progression of CKD although it was not statistically significant. Rather, as shown in Table 1, serum UA was lower in smokers, especially heavy smokers. Lower serum UA in smokers has been detected in previous studies (38, 39). Therefore, the role of hyperuricemia in the development of CKD in smokers also remains uncertain.

This cross-sectional observation has a critical limitation in considering causal associations. Furthermore, the limited number of the subjects, especially the small number of heavy smokers consuming one pack of cigarettes or more a day in the present study, may have somewhat skewed the study results. The lower values of serum LDLc, UA, and insulin observed in the heavy smoker in the present study should be confirmed in larger studies. Moreover, we analyzed the associations of current smoking habit and CKD and CVD risk factors, but not the effects of cumulative cigarette consumption on the development of CKD and CVD risk factors, which may give different results. In addition, the subjects were limited to male workers in the present study, and the results may differ in females since sex differences in the effects of smoking on CVD risk factors have been suggested by previous studies (40). Further studies should also be conducted in female subjects.

It can be concluded that elevated BP and serum GGT are significant characteristics of smokers showing CKD. Since the associations of BP and serum GGT with CKD in smokers are not considered to be entirely mediated by increased insulin resistance or chronic inflammation, further investigations to reveal the role of oxidative stress or serum TG in developing the associations are required.
